# Digital transformation and innovation output of manufacturing companies—An analysis of the mediating role of internal and external transaction costs

**DOI:** 10.1371/journal.pone.0296876

**Published:** 2024-01-19

**Authors:** Xiangpeng Meng, Xinshu Gong

**Affiliations:** School of Economics and Management, Shihezi University, Shihezi, Xinjiang, China; Lingnan University, The University of HongKong, HONG KONG

## Abstract

Digital transformation, based on digital technologies, has triggered economic growth in many industries and brought about production and service transformation in the manufacturing sector. As an important source of innovation output and a driving force for national economic development, it is of great significance to study the impact of digital transformation on innovation output in manufacturing companies. This study analyzes the effects of digital transformation on the quality, quantity, and overall innovation output of manufacturing companies from both the macro provincial-level digital transformation and micro enterprise-level digital transformation perspectives. Additionally, using data from manufacturing companies listed on the Shanghai and Shenzhen stock exchanges from 2012 to 2022, this study empirically tests the mechanism through which digital transformation affects innovation output from the perspectives of internal transaction costs and external transaction costs. The results show that digital transformation promotes overall improvement in innovation output of manufacturing companies and leads to improvements in both the quality and quantity of innovation output. Furthermore, the study finds that the effect of digital transformation on innovation output has a nonlinear characteristic under different levels of market competitiveness and market freedom. The mediation analysis reveals that the influence of digital transformation on innovation output can be attributed to the reduction of internal transaction costs and the enhancement of external transaction efficiency. In terms of digital policy formulation, it is necessary to coordinate the development of diverse and innovative digital infrastructure at the macro level with the micro-level ecosystems of enterprises, in order to reduce transaction costs within and outside innovative entities. Ultimately, it is essential for the government to foster a conducive free market environment that enhances transaction efficiency and timely regulates the excessive competition resulting from oligopolistic monopolies, thus maximizing the potential of digital transformation in promoting innovation output.

## 1. Introduction

With the rapid development of digital technologies such as big data, blockchain, cloud computing, and artificial intelligence, human economic society has entered a new era based on digital resources and centered around digitalization [1,2]. Driven by the dual forces of Chinese government policy guidance and practical development, both the macro-level digital infrastructure construction of cities and the micro-level digital transformation of enterprises have evolved from strategic foresight plans to inevitable choices driving high-quality economic development. According to the research findings of the “China Digital Economy Development White Paper (2021)”, by the end of 2020, the contribution of China’s broad digital industry to the scale of the economy accounted for 38.6% of that year’s GDP, and the industrial economic growth driven by digital transformation has become an important and undeniable force [3].

The manufacturing industry plays a pivotal role in driving the development of China’s real economy. Therefore, promoting innovation output of manufacturing enterprises is crucial for enhancing high-quality development in the manufacturing industry and sustaining development in China’s real economy.

As a result, at the policy-making and legal system levels [4], the Chinese government is guiding manufacturing enterprises in the transformation of manufacturing industry using new digital technologies [5]. It has created favorable environmental conditions for manufacturing companies to utilize new digital technologies in transforming manufacturing innovation output and creating enterprise value [6], while emphasizing the equal importance of “digital elements” along with traditional economic factors such as land, labor, and capital. Although the annual average growth rate of total patent applications for Chinese manufacturing companies is 16%, the proportion of invention patents that reflects the level of innovation quality has been declining at a rate of 35%. Moreover, in the critical technology fields of manufacturing key components and materials, the quality and quantity of innovation output from Chinese manufacturing enterprises are relatively low [7]. Therefore, can digital transformation, with its knowledge spillover effects and digital empowerment, promote the improvement of innovation output quality and quantity for manufacturing enterprises? Under the framework of transaction cost theory, the quality and quantity of innovation output depend not only on the macro-level environmental support and the micro-level input of innovation elements but also on the transaction costs that constrain the innovation results of manufacturing enterprises. Existing literature on digital transformation and innovation output mainly explores the impact of digital transformation on innovation output levels from the perspectives of resource complementarity, information processing, and dynamic capabilities, with limited research examining digital transformation from the angle of internal and external transaction costs. Therefore, this paper argues, from the dual perspectives of internal transaction costs and external transaction costs, the relationship between digital transformation, transaction costs, and innovation output. It also explains the dynamic impact relationship between digital transformation and innovation output under different market conditions. This provides some insights into understanding the inherent complexity of the impact of digital transformation on manufacturing enterprises’ innovation output and offers policy guidance for the government in formulating the direction of digital transformation policies.

## 2. Literature review and theoretical analysis

### 2.1 Digital transformation and innovation performance

Digital transformation, by optimizing the innovation factor structure [8,9], changing and even creating new production functions, can rationalize the allocation of resources for innovation input and effectively enhance the level of innovation output and firm innovation performance. Furthermore, with the free flow of data elements and the deep application of digital technologies, the rapid development of industrial digitization and digital industries, future digital transformation will promote cooperation among innovative organizations, transform traditional innovation models into collaborative platforms and ecosystems, and facilitate technological innovation in enterprises [10–13]. This will play an irreplaceable new role.

Based on existing research on digital transformation and innovation, this study argues that there are several reasons why digital transformation enhances firm innovation performance. Firstly, digital transformation alleviates transaction costs resulting from information asymmetry between trading parties. Digital transformation not only reduces information asymmetry between the innovation product market and the consumer market but also facilitates low-cost penetration of information, which can lower the cost of innovation trial and error and enhance regional innovation efficiency [12,14–16]. Additionally, innovation activities require significant resources in terms of manpower, funds, and time, while innovation outcomes and performance are inherently uncertain. The advantage of digital transformation in reducing the cost of innovation trial and error can alleviate innovation pressure [17] and simultaneously promote the quantity and quality of innovation output for firms or organizations. Moreover, the vast amount of information brought about by digital transformation enables manufacturing firms to perceive cutting-edge innovation technology changes in advance and respond to valuable digital technologies in the future [18]. This can enhance the technological responsiveness of manufacturing firms and further improve the quality of innovation output.

Secondly, digital transformation reduces transaction costs for firms to acquire innovation knowledge and external innovation resources. Compared to the non-digital era, where there were limitations on the internal resources of a single innovation entity [19], the R&D activities in the digital era require not only the resource input of individual innovators but also collaborative cooperation and value exchange along the industry chain based on the innovation outcomes. Therefore, effectively seeking external resources and information support, and reducing transaction costs associated with inter-organizational information exchange, contribute to an increase in the quantity of innovation for firms or organizations [20]. At the same time, the characteristics of digital technologies such as “addressability, perceptibility, communicability, storability, and traceability” can enhance the utility of digital technologies embedded in innovation platforms, thereby expanding and enhancing the ability of firms to collaboratively utilize innovative knowledge. Furthermore, the digital sensing capability endowed by digital transformation enables manufacturing firms to gather intelligence on innovation trends in the innovation market, accurately identify innovation opportunities and technological threats [21], and clarify the direction of future market innovation demands. Finally, in the open innovation network structure built upon digital transformation, the scale, efficiency, and integration of information flow will be significantly improved, which can help innovative enterprises find potential partners with whom to form strategic alliances [22], thus further unleashing the potential for innovation output.

Thirdly, digital transformation has changed the logic of competition in innovation activities among enterprises. Digital innovation platforms have transformed traditional R&D innovation models [23], broadened the boundaries of innovation between industries and within industries, and even altered the boundaries between firms and markets [24]. Digital transformation has also shifted traditional R&D activities from an internal development mode to open innovation activities embedded in social networks, thereby changing the competition in innovation activities from the traditional “winner takes all” logic to a “shared empowerment” logic. The “reprogrammability and associability” [25] of digital technologies facilitate collaboration among innovative organizations and reduce transaction costs in innovation cooperation. Therefore, digital innovation platforms facilitate the exchange and spillover effects of innovative knowledge in cross-regional cooperative organizations, enrich the breadth of knowledge for innovative organizations or companies, and expand the collaborative modes among cross-regional innovation entities, thus contributing to the improvement of innovation quality for businesses.

Based on the analysis above, this study proposes the following hypotheses regarding the relationship between digital transformation and innovation output for manufacturing companies:

H1: Digital transformation promotes the overall level of innovation output for manufacturing companies.
H1a: Digital transformation generally promotes the level of innovation quantity output for manufacturing companies.
H1b: Digital transformation generally promotes the level of innovation quality output for manufacturing companies.

### 2.2 Digital transformation, internal transaction costs, and innovation performance

Within internal company operations, digital transformation has a positive impact on innovation performance by reducing sales costs, financial costs, and management costs. From the perspective of internal business operations, digital transformation improves operational efficiency, effectively reduces financial and management costs, and the digital transformation platform saves sales expenses [26]. This allows companies to have more funds to support R&D activities, thus promoting innovation performance. Specifically, the widespread application of digital technologies such as big data, artificial intelligence, cloud platforms, and robotics optimizes traditional production, sales, and service processes within companies, effectively enhancing operational efficiency, reducing internal transaction costs, and ultimately improving innovation performance. Additionally, automation, business process improvements, and cost savings are significant components of digital transformation [27,28]. Moreover, technologies such as cloud computing, big data analytics, and smart products provide companies with more flexible production capabilities, reduce decision-making complexities for management when dealing with complex information systems.

Based on the aforementioned digital technologies and platforms, companies enhance the efficiency of information feedback in innovation input and output activities by adopting integrated production and service systems, intelligent supply chains, and online sales platforms. This creates valuable opportunities in the innovation market. Therefore, the lower internal transaction costs brought about by digital transformation become an important driver for improving innovation performance and outcomes.

Meanwhile, the specific assets required for innovation in the manufacturing industry represent sunk costs that cannot be easily monetized. Riordan and Williamson highlighted that asset specificity can serve as a measure of transaction costs in transaction cost economics [29]. R&D and innovation activities in the manufacturing sector often require highly specific physical assets, which have high acquisition costs. Due to their uniqueness and low versatility, if entrepreneurs change their direction or introduce an innovative product that is not accepted by the market, the specificity of asset investments in innovation becomes sunk costs [30]. The accumulated sunk costs resulting from trial and error in innovation further weaken the innovation intentions of the innovation entities. Therefore, this study proposes the hypothesis:

H2: At the corporate governance level, digital transformation reduces internal transaction costs by lowering sales costs, financial costs, management costs, and dedicated asset costs, thereby promoting the level of innovation output.

### 2.3 Digital transformation, external transaction costs, and innovation performance

Digital transformation can facilitate the improvement of innovation performance by reducing dispersed innovation relationships [31] and utilizing new financing tools [32]. It can help alleviate the increased transaction costs caused by financing constraints faced by enterprises and the uneven allocation of regional innovation resources, thereby promoting innovation performance enhancement. Search and recombination are important sources of increased innovation output in the innovation process. Digital transformation lowers search costs and enhances the innovation vitality of companies by facilitating the search and recombination of innovative knowledge. Knowledge recombination resulting from digital transformation leads to the generation of layered innovation knowledge, integration of innovation knowledge, and grafting of innovation knowledge. However, in internet platforms based on traditional information networks, valuable flows of innovative information generated by innovation entities often go unnoticed by the market and are not commercialized by downstream companies in the value chain, resulting in the loss of utility for valuable original innovation ideas. Digital transformation can enhance the transformation of various business processes based on innovation data collection, recognition, storage, and transformation [13], significantly improving the sharing of innovation value among different innovation entities and increasing the ability of different innovation enterprises to integrate into innovation networks, thereby reducing transaction costs associated with participating in innovation networks.

When R&D activities cannot be carried out due to insufficient funding, companies can utilize digital transformation to adjust the relationship between digital technology and information required by the market, standardize and structure information required by various markets, improve the availability of innovative information, and take the initiative to “push” valuable innovative information to the market [33], thereby enhancing the intangible asset value of the company. External investors are more willing to provide financial support to target companies based on the verifiability characteristics of digital data. This two-way exchange of data information significantly reduces the dispersion of innovation networks caused by geographical distances. Similarly, the bi-directional flow of information also plays an important role in alleviating insufficient liquidity and financing constraints [34].

Furthermore, at the macro level, digital transformation promotes the rational allocation of regional innovation resources, reduces external transaction costs for companies at the micro level, and enhances innovation performance. Digital transformation primarily optimizes the flow of innovation factors, improves the aggregation and integration of innovation infrastructure, facilitates information exchange of innovation activities and enables the value realization of innovation outcomes in the market, thus reducing transaction costs associated with traditional innovation value realization [17,35,36]. At the same time, the massive flow and high permeability of data resulting from digital transformation help accelerate the resolution of mismatches between different value attributes of innovation and different domains of innovation resources. This macro-level facilitation of micro-level innovation performance, in turn, intensifies the feedback loop of micro-level digital transformation on urban digital transformation, forming a mutually reinforcing closed-loop system. Therefore, with increased overall digital transformation, digital transformation promotes the enhancement of micro-level innovation capabilities by reducing external transaction costs for companies. Therefore, this study proposes the hypothesis:

H3: Digital transformation improves innovation performance for manufacturing companies by facilitating their integration into open innovation platforms, reducing the dispersion of innovation network relationships, and thereby lowering external transaction costs.

## 3. Research design, indicator construction, and data processing

### 3.1 Data sources and sample selection

This study primarily investigates the impact of digital transformation on innovation output efficiency in manufacturing enterprises. Due to the characteristics of a large number of manufacturing individual companies, long establishment periods, and relatively complete financial statements, the study focuses on manufacturing companies listed on the Shanghai and Shenzhen Stock Exchanges (A-share). Considering the impact of the 2008 global financial crisis, to ensure the credibility of regression results and hypothesis verification, the study selects A-share listed companies from 2012 to 2022 as the research sample. Patent application data are obtained from the China National Research Data Sharing Platform (CNRDS), and other research data are sourced from the CSMAR database, Wind database, and annual China Statistical Yearbook. Abnormal samples in non-normal listing status and critical missing data samples are excluded. Additionally, the principal variables of the sample are trimmed at the top and bottom 1%. In total, 10202 valid observations are obtained.

### 3.2 Variable definition and indicator construction

(1) Dependent variable

To measure the innovation performance of manufacturing companies, this study adopts the number of patent applications as an indicator of innovation output in the manufacturing industry. According to the definition provided by the China National Intellectual Property Administration and the Implementation Rules of the Patent Law, the number of invention patents, utility model patents, and design patents can all serve as indicators of enterprise patent applications. Among them, the total number of patent applications reflects the overall level of innovation resource input and innovation efficiency at the enterprise level. The number of invention patent applications reflects the “quality” feature of enterprise innovation performance. Utility model patents and design patents, as they are influenced by management’s innovation decision-making and protect the intellectual property rights of product structure and design rather than technical performance, reflect the “quantity” feature of enterprise innovation performance with relatively lower technical content compared to invention patent applications. Specifically, this study measures the overall level of firm innovation performance by taking the natural logarithm of the total number of patent applications plus 1, denoted as Invt. The quality feature of firm innovation performance is measured by taking the natural logarithm of the total number of invention patent applications plus 1, denoted as Invq. The quantity feature of firm innovation performance is measured by taking the natural logarithm of the geometric average of the total number of utility model patent applications and the total number of design patent applications plus 1, denoted as Invn and Inva, respectively.

(2) Independent variables

Based on the dimensions of digital platform application, digital infrastructure, digital industry development, digital business application, and digital development environment, a macro-level Digital Transformation Development Index (DTMA) is constructed. This index combines the important macro-factor of digital transformation with the micro-level enterprise digital transformation index.

To characterize the degree of micro-level digital transformation, the approach of Jiang et al. (2022) is followed [37]. The frequency of occurrence of digital-related vocabulary in the annual reports of all observed companies is extracted to construct the micro-level digital transformation indicator (DTMI). Additionally, to reduce statistical bias and the influence of non-digital word frequencies, judgmental words such as “none, ““without, “and “not” appearing in the statistical vocabulary are removed. The specific steps are as follows: From the four dimensions of digital technology application, digital strategy leadership, digital organizational empowerment, and digital information systems, Python is used to collect and analyze textual data on digital investment from the annual reports of listed companies. The digital transformation word frequency for each dimension is calculated for the sample companies, resulting in a word frequency table for each dimension of digital transformation. The calculated digital transformation indexes for the four dimensions are then transformed using logarithm, weighted with a 25% weight for each dimension, and combined to form the digital transformation index (DTMI). The digital transformation index constructed through this method objectively characterizes the degree of digital transformation in enterprises from the four dimensions of digital technology application, digital strategy leadership, digital organizational empowerment, and digital information systems.

(3) Mediating variable

For the measurement of internal transaction costs in manufacturing firms (ITC), sales costs, financial costs, internal operational costs, and innovation-specific asset costs are fitted, and the final results obtained were then multiplied by 100%. Sales costs (innercost1) are represented by the ratio of current sales expenses to current operating income. Financial costs (innercost2) are represented by the ratio of current financial expenses to current operating income. Internal operational costs (innercost3) are represented by the ratio of operating management expenses to operating income. Innovation-specific asset costs (innercost4) are represented by the proportion of investment in internal specific R&D assets to operating income. For the measurement of external transaction costs in manufacturing firms (OTC), governance technology expenditure in digital finance support input (digital finance coverage rate), investment in digital market environment (proportion of digital payments and e-commerce scale index), and government digital support expenditure (proportion of local fiscal digital technology expenditure) are fitted using an entropy method. OTC is the reverse indicator of external transaction costs.

(4) Control Variables

To ensure the reliability of the estimated coefficients, this study introduces control variables that reflect firm characteristics, as well as financial and governance structure characteristics. Firstly, firm characteristic control variables are considered. The difference between the sample year and the year of company establishment is used to calculate the firm’s age. Additionally, corporate governance structure variables are included. A dummy variable, dual, is introduced. If the positions of the chairman and CEO are combined, the variable is assigned a value of 1; otherwise, it is assigned a value of 0. The proportion of independent directors (ind) and the size of the board of directors (bs) are also controlled. Secondly, financial characteristic control variables are considered. Capital structure (leverage) is measured by the ratio of total liabilities to total assets. The fixed asset ratio (fixa) is calculated as the ratio of net fixed assets to total assets. Profitability of manufacturing companies is measured by the ratio of net profit to total assets (roa) and the natural logarithm of profit (lpro). The explanation of variables can be found in Table 1. Descriptive statistics for the main variables are presented in Table 2.

**Table 1:** Explanation of variables.

Variable Name	Variable symbols	Variable construction method
**Dependent Variable**		
Overall Innovation Performance	Invt	LN(1 + total number of patent applications for the year)
Innovation Output Quality	Invq	LN(1 + number of invention patent applications for the year)
Innovation Output Number	Invn, lnva	LN(1 + number of utility model patent applications for the year), LN(1 + number of design patent applications for the year)
**Independent Variable**		
Digital Transformation	DT	It includes the provincial-level macro digital transformation index (dtma) and the micro-level enterprise digital transformation index (dtmi). These two components together form the total digital transformation index (dt) with a 50% weight assigned to each.
**Mediating Variable (tc)**		
Internal Transaction Costs	Itc	fitted with sales costs, financial costs, management costs, and innovation-specific asset costs.
External Transaction Costs	Otc	fitted with financing constraint costs and regional innovation resource input costs from local government
**Control Variable**		
Proportion of Independent Directors	Ind	Number of independent directors divided by the total number of board members.
Company Age	Age	Difference between the current year and the year of establishment.
Fixed Asset Ratio	Fixa	Reflects the ratio of net fixed assets to total assets.
Board Size	Bs	Natural logarithm of the number of board members
Dual Role	Dual	If the roles of the chairman and CEO are combined, the variable value is 1, otherwise 0.
Debt-to-Asset Ratio	Lev	Measures the capital structure by dividing total liabilities by total assets.
Return on Assets	Roa	Ratio of net profit to total assets.
Operating Profit	lpro	Natural logarithm of operating profit.
Regional GDP	lgdp	Natural logarithm of regional GDP.
Year Fixed Effect	Year FE	Dummy variable for years.
Industry Fixed Effect	Industry FE	Dummy variable for industries.
**Moderating Variable**		
Market Competition Level	Mh	Square sum of the ratio of the company’s annual revenue to the total industry revenue (Mh).
Market freedom Level	Mf	Marketization index of various provinces.

**Table 2:** Descriptive statistics analysis.

Variables	Observations	Mean	SD	Max	Min
Invt	10202	3.8866	2.3145	13.0376	0.0000
Invq	10202	1.7223	1.0947	6.5669	0.0000
Invn	10202	1.9086	1.3249	6.0263	0.0000
DT	10202	2.5235	0.6630	5.5397	0.2895
Dtma	10202	2.4487	0.9444	6.1349	0.0000
Dtmi	10202	2.5982	0.7843	8.2351	0.0399
Itc	10202	15.2557	6.2725	50.7648	6.7252
Otc	10202	1.7534	0.8337	5.4885	-1.7797
Ind	10202	0.3660	0.0498	0.5657	0.3300
Age	10202	2.5989	0.4311	3.3495	1.1043
Fixa	10202	0.3035	0.1865	0.8491	0.0025
Bs	10202	2.1220	0.1867	2.6804	1.5930
Dual	10202	0.2921	0.4554	1.0000	0.0000
Lev	10202	0.4998	0.1672	0.9925	0.0071
Roa	10202	-0.1710	0.0697	0.1916	-0.2755
Lpro	10202	7.8323	1.1354	10.8610	2.8046
Lgdp	10202	16.2948	0.6352	19.4567	15.7329

### 3.3 Econometric model specification

A Hausman test was conducted to examine the relationship between the Digital Transformation Index of enterprises (Dtmi), provincial-level Digital Transformation Index (Dtma), and innovation output (INV), including both the quantity of innovation output (Invn) and its quality (Invq). The results strongly reject the null hypothesis, indicating that a fixed-effects model should be used to test the baseline relationship. The fixed-effects model can control for unobserved factors and mitigate endogeneity issues. Based on this, following Lee et al.’s (2010) study, a fixed-effects model incorporating time and individual effects was constructed. The model is as follows:

To examine the overall effect of digital transformation on firm innovation performance, as well as the quantity and quality effects of digital transformation on firm innovation output, the econometric baseline model for digital transformation and innovation performance constructed in this study is as follows:

$$Inv_{i,t,j}=\alpha_{0}+\alpha_{1}DT_{i,t}+\alpha_{2}\sum Control_{i,t}+\mu_{i}+\delta_{i}+\varepsilon_{i,t}$$ (1)

The dependent variable, innovation performance (*Inv_i,t,j_*), represents the innovation performance of research sample i in year t. The index j represents three sub-dimensions of innovation performance: innovation output level (Invt), innovation quality level (Invq), and innovation quantity level (Invn). The explanatory variable, digital transformation (*DT_i,t_*), is represented by the arithmetic average of the regional macro-level digital transformation level and the firm micro-level digital transformation level. In addition, a series of control variables (∑*Control_i,t_*) are considered, such as fixed asset ratio, debt-to-asset ratio, return on assets, operating profit, etc.

Following Hausman tests on innovation output, digital transformation, and other related variables, the null hypothesis is rejected at the 1% level. Therefore, this study employs firm-specific fixed effects (μi) and year*industry fixed effects (*δ_i_*) in the analysis. The year industry fixed effects control for unobservable factors that vary over time at the industry level. The error term (*ε_i,t_*) captures other unconsidered influencing factors.

Furthermore, to verify the potential mediating mechanism of internal and external transaction costs between digital transformation and firm innovation output, an intermediary effect model is constructed based on the baseline regression model. Specifically, the regression coefficient of digital transformation (DT) on total innovation output is tested, followed by constructing the regression equation for digital transformation (DT) on manufacturing firms’ transaction costs (TC). Transaction costs (TC) include two aspects: internal transaction costs (ITC) and external transaction costs (OTC). Finally, the regression equation for the relationship among digital transformation (DT), internal and external transaction costs (TC), and total innovation output (Invt) is constructed. The specified intermediary effect model is as follows:

$${\mathrm{TC}}_{i,t,j}=\alpha_{0}+\alpha_{1}DT_{i,t}+\alpha_{2}\sum Control_{i,t}+\mu_{i}+\delta_{i}+\varepsilon_{i,t}$$ (2)

$$Invt_{i,t,j}=\alpha_{0}+\alpha_{1}DT_{i,t}+\alpha_{2}\mathrm{TC}+\alpha_{3}\sum Control_{i,t}+\mu_{i}+\delta_{i}+\varepsilon_{i,t}$$ (3)

To explore the potential nonlinear relationship between digital transformation and firm innovation performance levels under different market environment conditions, this study constructs a panel threshold model for digital transformation and firm innovation performance in different market environments:

$$\begin{array}{l} Invt_{i,t,j}=\beta_{0}+\beta_{1}DT_{i,t}\times Th(Market\leq \theta )+\beta_{2}DT_{i,t}\times Th(Market>\theta )\\ +\beta_{3}\sum Control_{i,t}+\mu_{i}+\delta_{i}+\varepsilon_{i,t} \end{array}$$ (4)

Where market competitiveness (MH) and market freedom (MF) serve as threshold variables (th) in the model.

## 4. Analysis of the overall effect of digital transformation on innovation output of manufacturing companies

The empirical results displayed in Table 3 indicate a significant positive relationship between the coefficient of overall digital transformation and innovation output in all four regression models, both with and without control variables. This suggests that the digital transformation, as constructed in this study, overall promotes the innovation output, quality, and quantity levels of manufacturing companies listed on China’s A-share market. In terms of economic significance, taking column (2) as an example, the mean of overall innovation output (lnvtol) is 3.8866. An increase of 1% in the level of overall digital transformation, composed of macro-level digital transformation and enterprise-level digital transformation, is associated with an approximate increase of 2.018 units in the overall innovation level of local manufacturing companies (i.e., 3.8866 × 0.5194 = 0.17). Mechanism analysis indicates that digital transformation enhances the macro-level support of digital environments, reduces the search and recombination costs for innovation, and improves the integration and grafting effects of innovative knowledge. This allows digital technologies to be accurately targeted at companies with innovation potential, and digital platforms alleviate the challenges faced by manufacturing companies in terms of innovation funding shortages and decentralized innovation network relationships.

**Table 3:** Baseline regression results.

Variables	(1)	(2)	(3)	(4)	(5)
	Invt	Invt	Invq	Invn	Inva
DT	0.5463***	0.5194***	0.3582***	0.1837***	0.1799***
	(11.8916)	(11.7657)	(13.4514)	(7.8636)	(9.5089)
Ind		-0.0347	0.2662	0.1265	-0.2573
		(-0.0491)	(0.6433)	(0.3538)	(-0.8282)
Age		0.3868	0.0216	0.1916	0.1617
		(1.6241)	(0.1596)	(1.4886)	(1.5484)
Fixa		-0.1568	-0.0391	-0.0727	-0.0489
		(-0.7750)	(-0.3482)	(-0.6525)	(-0.5543)
Bs		0.4715**	0.3022***	0.3135**	0.1014
		(2.0794)	(2.6752)	(2.4477)	(0.9643)
Dual		0.0102	-0.0059	-0.0261	0.0187
		(0.1543)	(-0.1687)	(-0.6675)	(0.6292)
Lev		-0.5105***	-0.1458**	-0.2992***	-0.2275***
		(-4.7934)	(-2.5554)	(-4.7177)	(-4.7469)
Roa		2.8056***	1.3817***	1.3608***	0.9912***
		(4.3366)	(3.8822)	(3.8404)	(3.7088)
Lpro		0.2826***	0.1146***	0.1518***	0.1115***
		(8.7510)	(6.8231)	(7.9161)	(8.0269)
Lgdp		0.0588*	0.0099	0.0444**	0.0318**
		(1.7764)	(0.5454)	(2.2790)	(2.1445)
Constant term	1.1767***	-2.8065***	-0.9960*	-1.9849***	-1.0328**
	(10.9898)	(-2.7931)	(-1.7587)	(-3.5837)	(-2.3294)
N	10202	10202	10202	10202	10202
R-Squared	0.3023	0.3208	0.2081	0.3039	0.2463
Adj. R-Squared	0.3016	0.3194	0.2065	0.3026	0.2448
Year FE	Yes	Yes	Yes	Yes	Yes
Control FE	No	Yes	Yes	Yes	Yes

Notes: t statistics in parentheses

* p < 0.1

** p < 0.05

*** p < 0.01. These are consistent in the following tables.

Additionally, digital transformation reduces internal transaction costs by lowering sales costs, financial costs, management costs, and innovation-specific asset costs, thereby promoting innovation output levels of manufacturing companies. Furthermore, according to the baseline regression results, the control variable of debt-to-asset ratio (lev) displays a significantly negative influence on innovation performance in the manufacturing industry at the 1% level, indicating that higher levels of debt are detrimental to improving innovation performance. Other control variables such as return on assets (roa) and corporate profits (lpro) are positively correlated with innovation performance in the manufacturing industry at the 1% level, highlighting the importance of sound operational conditions as a necessary condition for conducting innovative activities in the manufacturing industry. Moreover, to explore the relationship between digital transformation and innovation quality and quantity in the manufacturing industry, this study uses innovation quality index (Invq) and innovation quantity index (Invn) as dependent variables in columns (3) to (5) of the regression results. The findings demonstrate that digital transformation significantly enhances not only the overall innovation performance of manufacturing companies but also the quality and quantity of their innovation output. Therefore, hypotheses H1, H1a, and H1b are all supported.

## 5. Further examination

### 5.1 Mechanism test based on internal and external transaction costs

To further investigate the specific mechanism of how digital transformation affects innovation output in manufacturing companies, this study empirically examines the mediating variables of internal transaction costs and external transaction costs. Table 4 reflects the impact of internal transaction costs and external transaction costs on the relationship between digital transformation and innovation output in manufacturing companies. From columns (1), (2), and (3), it can be observed that the influence of digital transformation on internal transaction costs in manufacturing companies is significantly negative. Similarly, the coefficient of internal transaction costs on innovation output in manufacturing companies is also significantly negative, indicating a significant mediating effect of internal transaction costs between digital transformation and innovation output in manufacturing companies. The overall digital transformation constructed at the macro and micro levels reduces internal operating costs, management costs, advertising expenses, sales expenses, and other expenses, thereby decreasing internal transaction costs, improving internal transaction efficiency, and enhancing the ability of companies to generate innovative output.

**Table 4:** Results of mediation effects testing.

Variables	(1)	(2)	(3)	(4)	(5)
	Invt	Itc	Invt	Otc	Invt
DT	0.5194***	-0.6486***	0.4067***	0.0914***	0.3587***
	(11.7657)	(-4.6878)	(11.5246)	(7.2511)	(9.9726)
Ind	-0.0347	0.5278	0.0570	-0.1462	0.2225
	(-0.0491)	(0.2638)	(0.0995)	(-0.6289)	(0.3734)
Age	0.3868	-1.2451	0.1704	-0.2340***	0.7984***
	(1.6241)	(-1.6297)	(0.8951)	(-2.7607)	(3.7593)
Fixa	-0.1568	-0.0122	-0.1590	-0.3327***	0.4285**
	(-0.7750)	(-0.0179)	(-0.9833)	(-4.5940)	(2.5161)
Bs	0.4715**	-1.2174*	0.2599	0.2293***	0.0682
	(2.0794)	(-1.6726)	(1.4907)	(2.9364)	(0.3463)
Dual	0.0102	-0.0151	0.0076	0.0563**	-0.0888
	(0.1543)	(-0.0756)	(0.1340)	(2.4709)	(-1.5323)
Lev	-0.5105***	0.4581	-0.4309***	0.3249***	-1.0821***
	(-4.7934)	(1.1187)	(-4.9530)	(7.5383)	(-10.9891)
Roa	2.8056***	-20.4805***	-0.7538	-1.9794***	6.2875***
	(4.3366)	(-10.6331)	(-1.3980)	(-5.6570)	(9.2080)
Lpro	0.2826***	-0.4783***	0.1995***	0.3803***	-0.3864***
	(8.7510)	(-4.4895)	(7.5273)	(25.9742)	(-11.6061)
Lgdp	0.0588*	-0.1949*	0.0250	-0.0700***	0.1819***
	(1.7764)	(-1.9411)	(0.9296)	(-5.4852)	(6.6052)
Itc			-0.1738***		
			(-31.8882)		
Otc					1.7591***
					(26.9095)
N	10202	10202	10202	10202	10202
R-Squared	0.3208	0.1937	0.5612	0.3914	0.5411
Adj. R-Squared	0.3194	0.1921	0.5603	0.3902	0.5402
Year FE	Yes	Yes	Yes	Yes	Yes
Control FE	Yes	Yes	Yes	Yes	Yes

From columns (1), (2), and (5) in Table 4, it can be seen that digital transformation has a significantly positive impact on the efficiency of external transactions in manufacturing companies. Simultaneously, the improvement in external transaction efficiency also has a significant promoting effect on innovation output. This indicates that digital transformation enhances the integration of manufacturing companies into social innovation networks, reduces search and innovation recombination costs in the innovation process, and consequently lowers external transaction costs for manufacturing companies. This strengthens the data capital advantage of manufacturing companies, improves the overall operational efficiency of innovation output, reduces communication and decision-making costs related to innovation for manufacturing companies, and promotes the development of innovation quality and quantity. Based on the analysis above, hypotheses H3 and H4 are validated.

### 5.2 Analysis of threshold effects based on market competitiveness and marketization level

The baseline empirical results in the previous findings indicate that with different degrees of digital transformation, it has a significant positive effect on overall innovation output, as well as the quality and quantity of innovation output in manufacturing enterprises. However, under different market structures and competitive environments, is the impact of digital transformation on innovation output in manufacturing companies different? To verify the effects caused by market competitiveness(Industry competitiveness) and market freedom level, we examine the influence of market competitiveness and market freedom level on the relationship between digital transformation and innovation output.

In theory, in industries with varying levels of competitiveness and marketization, the impact of digital transformation on innovation output in the manufacturing industry may exhibit nonlinear characteristics due to the different levels of competition and marketization. Similar studies have shown that the intensity of digital transformation in promoting innovation efficiency of Chinese firms varies under different external conditions. For example, the differentiation in period cost rates can affect the intensity of digital transformation in enhancing the quantity and efficiency of technological innovation in agricultural enterprises [38]. Similarly, the differing levels of R&D intensity also influence the relationship between investment and innovation output in high-tech enterprises [39]. However, it is worth noting that the heterogeneity of environmental conditions leads to significant variations in the results when digital transformation is applied to different research subjects, indicating that the results are not universally applicable. Therefore, we employ a panel threshold regression model for empirical testing. In Table 5, columns (1)-(3) are respectively using market competitiveness level (mh) and market freedom (mf) as threshold variables to represent the market competitiveness and market freedom. Market competitiveness is obtained by summing the square accumulation of the ratio of the company’s main business income and the book value of the owner’s equity to the company’s market share in the industry.

**Table 5:** Regression results of the threshold model.

Variables	(1)	(2)	(3)
	Mh1	Mh2	Mf
Threshold(k)	0.3988	1.1893	2.9849
Th#DT(Th≤k)	0.5400***	0.5390***	0.2271***
	(17.2739)	(17.2541)	(7.5366)
Th#DT(Th>k)	0.4935***	0.4869***	0.6863***
	(15.5743)	(14.9993)	(23.4830)
Ind	-0.0427	-0.0130	-0.1445
	(-0.0906)	(-0.0276)	(-0.3251)
Age	0.3756***	0.3651***	0.4959***
	(2.6807)	(2.6033)	(3.7545)
Fixa	-0.1458	-0.1419	-0.0332
	(-1.0338)	(-1.0057)	(-0.2494)
Bs	0.4653***	0.4678***	0.2757*
	(3.0457)	(3.0622)	(1.9127)
Dual	0.0152	0.0132	-0.0302
	(0.3163)	(0.2747)	(-0.6670)
Lev	-0.5065***	-0.5040***	-0.6319***
	(-5.1868)	(-5.1601)	(-6.8586)
Roa	2.7592***	2.7339***	3.0306***
	(6.2865)	(6.2228)	(7.3273)
Lpro	0.2837***	0.2828***	0.0430*
	(12.8905)	(12.8512)	(1.9542)
Lgdp	0.0587**	0.0572**	0.0899***
	(2.0342)	(1.9818)	(3.3048)
Constant term	-1.2185	-1.1755	0.4571
	(-1.5633)	(-1.5076)	(0.6205)
N	10202	10202	10202
R-Squared	0.3215	0.3215	0.3969
Adj. R-Squared	0.1837	0.1837	0.2744
Year FE	Yes	Yes	Yes
Control FE	Yes	Yes	Yes

The threshold regression results in Table 5 indicate that the impact of digital transformation on innovation output in the manufacturing industry exhibits a pattern of initial promotion followed by diminishing marginal effects as industry competitiveness increases. In other words, overall, digital transformation enhances innovation output in manufacturing companies, but the promoting effect on innovation output weakens as the level of digital transformation increases. Specifically, in column (1) using market competitiveness index 1 (Mh1) as the threshold variable, when the threshold variable Lehner index is below 0.3988, the impact coefficient of digital transformation on innovation output in manufacturing companies is 0.54; when the threshold variable Lehner index is above 0.3988, the impact coefficient of digital transformation on innovation output changes to 0.4935.

To ensure robustness of the results, this study constructs MH2 using the ratio of market share of the top ten firms to market share of the sample firms in their respective sub-industries, which also demonstrates diminishing marginal effects. Similarly, to examine the relationship between marketization level and the impact of digital transformation on innovation output, column (2) uses market freedom as the threshold variable. The coefficients of digital transformation on innovation output in manufacturing companies are 0.2271 and 0.6863 on either side of the threshold value of 2.9849. Thus, it can be concluded that the intensity of the impact of digital transformation on innovation differs under different market environments. In an environment with low market freedom restricted by government policies and laws, the promotion effect of digital transformation on innovation output is significantly reduced, to the extent that manufacturing companies are unable to effectively leverage the positive impact of digital transformation. However, in an environment with high market freedom, the promoting effect of digital transformation on innovation output in manufacturing companies is significantly enhanced. Therefore, the hypothesis of threshold effects of digital transformation on innovation output in manufacturing companies under different industry competitiveness and marketization conditions is supported. Overall, these findings highlight the importance of considering industry competitiveness and marketization level when examining the impact of digital transformation on innovation output in the manufacturing sector.

### 5.3 Robustness tests

1. Controlling for Multidimensional Fixed Effects

Considering the impact of differences in various sub-industries and regions in the manufacturing sector on innovation output, this study performs robustness tests by adding multidimensional fixed effects to the regression results of the impact of digital transformation on internal and external transaction costs and innovation output. The multidimensional fixed effects include industry fixed effects, province fixed effects, individual fixed effects, and industry and region fixed effects. Table 6 presents the robust regression results of the impact of digital transformation on innovation output, controlling for multidimensional fixed effects.

**Table 6:** Threshold model with multidimensional fixed effects.

Variables	(1)	(2)	(3)	(4)
	Invt	Invq	Invn	Inva
DT	0.5233***	0.3576***	0.1873***	0.1829***
	(12.1515)	(13.6562)	(8.0792)	(9.9187)
Ind	0.1335	0.3220	0.1929	-0.1721
	(0.1933)	(0.7953)	(0.5477)	(-0.5676)
Age	0.2722	-0.0034	0.1421	0.0933
	(1.2744)	(-0.0279)	(1.2465)	(0.9853)
Fixa	-0.1176	-0.0138	-0.0346	-0.0419
	(-0.6097)	(-0.1253)	(-0.3215)	(-0.5055)
Bs	0.4813**	0.2857**	0.3046**	0.1154
	(2.2252)	(2.5504)	(2.5344)	(1.1548)
Dual	0.0145	-0.0043	-0.0220	0.0187
	(0.2290)	(-0.1270)	(-0.5944)	(0.6576)
Lev	-0.4982***	-0.1316**	-0.2971***	-0.2282***
	(-4.7982)	(-2.3258)	(-4.8370)	(-4.9163)
Roa	2.8778***	1.4056***	1.3739***	1.0195***
	(4.5738)	(3.9759)	(4.0001)	(3.9265)
Lpro	0.3007***	0.1154***	0.1631***	0.1225***
	(9.9686)	(7.0699)	(9.6149)	(9.2637)
Lgdp	0.0553*	0.0082	0.0426**	0.0305**
	(1.6730)	(0.4544)	(2.2365)	(2.0488)
N	9950	9950	9950	9950
R-Squared	0.7863	0.7120	0.7751	0.7793
Adj. R-Squared	0.7485	0.6611	0.7354	0.7403
Hd FE	Yes	Yes	Yes	Yes

Notes: Hd FE stands for high-dimension fixed effects. These are consistent in the following tables.

Table 7 reports the robust empirical results of the impact of digital transformation on internal and external transaction costs, as well as innovation output, after controlling for multidimensional fixed effects. The results are consistent with the previous regression results, which indicate that digital transformation affects innovation output by altering internal and external transaction costs.

**Table 7:** Results of the mechanism test for internal and external transaction costs.

Variables	(1)	(2)	(3)	(4)	(5)
	Invt	Itc	Otc	Invt	Invt
DT	0.5233***	-0.6783***	0.0922***	0.4051***	0.3668***
	(12.1515)	(-4.9866)	(7.2520)	(11.8377)	(10.4285)
Ind	0.1335	0.2084	0.0028	0.1698	0.1287
	(0.1933)	(0.1067)	(0.0117)	(0.2995)	(0.2102)
Age	0.2722	-1.0004	-0.1911**	0.0978	0.5967***
	(1.2744)	(-1.4734)	(-2.1322)	(0.5743)	(2.9371)
Fixa	-0.1176	-0.2936	-0.3058***	-0.1688	0.4016**
	(-0.6097)	(-0.4439)	(-4.1192)	(-1.0951)	(2.3565)
Bs	0.4813**	-1.1829*	0.1674**	0.2751	0.1971
	(2.2252)	(-1.7149)	(2.0372)	(1.6190)	(0.9817)
Dual	0.0145	-0.0081	0.0762***	0.0131	-0.1149**
	(0.2290)	(-0.0421)	(3.2035)	(0.2407)	(-2.0330)
Lev	-0.4982***	0.4259	0.3313***	-0.4240***	-1.0607***
	(-4.7982)	(1.0598)	(7.6874)	(-4.9086)	(-10.8803)
Roa	2.8778***	-20.4866***	-1.8839***	-0.6925	6.0766***
	(4.5738)	(-10.8965)	(-5.4259)	(-1.3276)	(9.0833)
Lpro	0.3007***	-0.4884***	0.3799***	0.2156***	-0.3444***
	(9.9686)	(-5.1786)	(25.7323)	(8.5272)	(-9.5938)
Lgdp	0.0553*	-0.1997**	-0.0728***	0.0205	0.1789***
	(1.6730)	(-2.0112)	(-5.8773)	(0.7569)	(6.3354)
Itc				-0.1743***	
				(-32.4183)	
Otc					1.6980***
					(25.1557)
N	9950	9950	9950	9950	9950
R-Squared	0.7863	0.6597	0.8202	0.8622	0.8528
Adj. R-Squared	0.7485	0.5996	0.7884	0.8379	0.8268
Hd FE	Yes	Yes	Yes	Yes	Yes

2. Robustness Test of Subsample

We exclude samples where manufacturing companies have zero patent applications. The operating scope of Chinese A-share listed companies includes manufacturing sub-industries with low-end production, high pollution, and high energy consumption, which tend to have lower innovation outputs. Considering that some manufacturing companies have zero patent applications during the sample period, these companies are more inclined to engage in digital transformation in order to obtain analyst attention and government policy subsidies. To eliminate the interference caused by rent-seeking behavior from these manufacturing companies, we exclude companies with consistently zero patent applications. The regression results are reported in Table 8. After excluding manufacturing companies that have never applied for patents, it is found that digital transformation (DT) still has a significant promoting effect on both the quality and quantity of innovation output in manufacturing companies. This is consistent with the baseline regression results reported earlier.

**Table 8:** Robustness test regression results.

Variables	(1)	(2)	(3)	(4)
	Invt	Invq	Invn	Inva
DT	0.4922***	0.3300***	0.1498***	0.1646***
	(11.6342)	(12.6722)	(6.6562)	(8.8431)
Ind	0.3608	0.4061	0.5261	0.1353
	(0.5201)	(1.0244)	(1.5272)	(0.4444)
Age	0.1699	-0.0473	0.1143	0.0748
	(0.7923)	(-0.3829)	(1.0142)	(0.7771)
Fixa	-0.1578	0.0177	-0.0845	-0.0579
	(-0.8206)	(0.1600)	(-0.7864)	(-0.6869)
Bs	0.5909***	0.2532**	0.3671***	0.1723*
	(2.7080)	(2.2362)	(3.0830)	(1.7048)
Dual	0.0383	-0.0047	-0.0189	0.0310
	(0.5978)	(-0.1432)	(-0.5080)	(1.0548)
Lev	-0.3989***	-0.0967*	-0.2531***	-0.2005***
	(-3.8623)	(-1.6833)	(-4.1237)	(-4.1660)
Roa	2.6850***	1.2569***	1.1415***	0.9429***
	(4.2977)	(3.6061)	(3.2662)	(3.4594)
Lpro	0.2957***	0.0999***	0.1537***	0.1225***
	(9.8843)	(6.1340)	(9.1392)	(9.1087)
Lgdp	0.0522	0.0058	0.0294	0.0255*
	(1.5603)	(0.3029)	(1.5089)	(1.6652)
N	9569	9175	8848	9000
R-Squared	0.7858	0.6996	0.7752	0.7756
Adj. R-Squared	0.7468	0.6431	0.7317	0.7328
Hd FE	Yes	Yes	Yes	Yes

### 5.4. Endogeneity treatment

To address the endogeneity and omitted variable issues that may exist in the relationship between digital transformation and the quality and quantity of innovation output in the manufacturing sector, this study adopts instrumental variable estimation. The numbers of fixed-line telephones per household (IV1) and township-level post offices in each region at the macro level in 1990 (IV2) are used as instrumental variables. The number of fixed-line telephones per household and township-level post offices in the 1990s reflect the level of information and communication technology (ICT), satisfying the relevance requirement for digital technologies. Additionally, with the development of digital technologies, the impact of fixed-line telephones and township-level post offices on innovation output has rapidly diminished, meeting the exogeneity assumption of instrumental variables. To address the issue of missing time-series data in cross-sectional data, we follow the empirical method proposed by Nunn and Qian to construct an interactive term [40] between the local government’s investment in information technology infrastructure and the number of fixed-line telephones per hundred households in the 1990s as an instrumental variable for digital transformation. The instrumental variable (IV) approach utilized in this study requires the instrument to be correlated with the error term and to move in the same direction as the error term. Additionally, it requires that the degree of endogeneity of the instrumental variable is smaller than the endogeneity of the endogenous explanatory variable. The ultimately selected instrumental variable exhibits weaker endogeneity, resulting in a narrower bilateral interval that encompasses the true coefficient [41]. Therefore, the handling of endogeneity between digital transformation and innovation output in this study satisfies the basic assumptions of the IV approach.

Moreover, when constructing the digital transformation index, this study considers not only the micro-level index of digital transformation at the firm level but also the macro-level index of digital transformation at the regional level. It is evident that individual innovative firms alone do not have the capability to influence provincial-level digital infrastructure development and the diffusion of digital capabilities. Thus, both the construction of the digital transformation index and the development of the instrumental variable approach in this study take into account the issues arising from the endogeneity of digital transformation at the micro-level of enterprises, ensuring the reliability of the regression results.

Table 9 presents the regression results using the instrumental variable approach. After accounting for the endogeneity between digital transformation and innovation output, columns (1)-(3) show that the coefficient of digital transformation remains significantly positive, indicating that digital transformation significantly promotes both the total innovation output and the quality and quantity of innovation output. In columns (4)-(5), the results demonstrate that internal transaction costs have a significant negative effect on innovation output in the manufacturing sector, while external transaction efficiency has a significant positive effect. Moreover, the estimated coefficient of digital transformation decreases compared to the regression results in column (1), suggesting that internal and external transaction costs play a partial mediating role between digital transformation and innovation output. This aligns with the results of the mechanism analysis on mediation effects presented earlier.

**Table 9:** Instrumental variable estimation results.

Variables	(1)	(2)	(3)	(4)	(5)
	Invt	Invq	Invn	Invt	Invt
DT (IV)	1.2619***	0.5085**	0.5767**	0.8472**	0.7258**
	(3.3356)	(2.5101)	(2.5697)	(2.4436)	(2.2379)
Ind	-0.0283	0.2421	0.0978	-0.0788	0.2598
	(-0.0542)	(0.8534)	(0.3330)	(-0.1646)	(0.6016)
Age	0.3057*	0.0078	0.1441	0.0088	0.7559***
	(1.8252)	(0.0853)	(1.4827)	(0.0572)	(5.2355)
Fixa	-0.1516	-0.0423	-0.0521	-0.0153	0.4038***
	(-0.9954)	(-0.5079)	(-0.5858)	(-0.1075)	(3.1372)
Bs	0.4677***	0.2965***	0.3014***	0.4077***	0.0869
	(2.8505)	(3.5868)	(3.1234)	(2.7834)	(0.6134)
Dual	0.0202	-0.0027	-0.0174	0.0521	-0.0812*
	(0.3904)	(-0.1009)	(-0.5603)	(1.0918)	(-1.9178)
Lev	-0.4652***	-0.1331**	-0.2729***	-0.2812***	-1.0442***
	(-4.4685)	(-2.4478)	(-4.4701)	(-2.9026)	(-11.4672)
Roa	2.5779***	1.3521***	1.2178***	1.2670***	6.1459***
	(5.0166)	(4.8193)	(4.1734)	(2.6670)	(12.0262)
Lpro	0.2632***	0.1113***	0.1431***	0.2743***	-0.3801***
	(9.2520)	(7.5562)	(8.6136)	(10.3809)	(-15.0684)
Lgdp	0.0454	0.0099	0.0361*	0.0827***	0.1719***
	(1.3922)	(0.5739)	(1.8809)	(2.7714)	(6.4205)
Itc				-0.0537***	
				(-11.9538)	
Otc					1.7184***
					(30.8424)
N	9944	9944	9944	9681	9944
R-Squared	0.2794	0.2066	0.2714	0.3793	0.5340
Adj. R-Squared	0.1528	0.0672	0.1434	0.2717	0.4521

Furthermore, this study includes both instrumental variables IV1 and IV2 and the overall digital transformation index in the regression equation. If the regression coefficients of IV1 and IV2 on the dependent variable, innovation output, are not statistically significant, it strengthens confidence in the exogeneity of the instrumental variables IV1 and IV2. The estimation results are shown in Table 10, with both IV1 and IV2 instrumental variables exhibiting coefficients that are statistically significant at a level greater than 10%, indicating the exogeneity of the instrumental variables. This demonstrates the lack of correlation between the instrumental variables and innovation output, and confirms that the overall digital transformation index significantly promotes firm-level innovation output, consistent with the earlier findings.

**Table 10:** Estimation results of instrumental variable exogeneity.

Variables	(1)	(2)	(3)
	Invt	Invt	Invt
DT	0.5137***	0.5150***	0.5145***
	(11.6775)	(11.6825)	(11.7222)
IV1	0.0135		0.0147*
	(1.2735)		(1.6541)
IV2	0.0019	0.0072	
	(0.2506)	(1.0998)	
Ind	-0.0404	-0.0148	-0.0468
	(-0.0568)	(-0.0208)	(-0.0659)
Age	0.3689	0.3763	0.3703
	(1.5520)	(1.5832)	(1.5573)
Fixa	-0.1504	-0.1516	-0.1514
	(-0.7449)	(-0.7494)	(-0.7494)
Bs	0.4671**	0.4714**	0.4667**
	(2.0512)	(2.0755)	(2.0485)
Dual	0.0130	0.0138	0.0121
	(0.1946)	(0.2081)	(0.1831)
Lev	-0.5119***	-0.5137***	-0.5110***
	(-4.8011)	(-4.8209)	(-4.7949)
Roa	2.7697***	2.7780***	2.7745***
	(4.2688)	(4.2846)	(4.2773)
Lpro	0.2800***	0.2813***	0.2801***
	(8.7051)	(8.6953)	(8.7199)
Lgdp	0.0575*	0.0583*	0.0576*
	(1.7404)	(1.7651)	(1.7404)
Constant term	-2.7881***	-2.8130***	-2.7845***
	(-2.7780)	(-2.7995)	(-2.7728)
N	10202	10202	10202
R-Squared	0.3214	0.3210	0.3213
Adj. R-Squared	0.3199	0.3196	0.3199

## 6. Conclusion and policy implications

Based on the data of manufacturing companies listed on China’s A-share market, this study constructs the level of digital transformation through the innovative approach of combining “macro provincial-level digitalization indicators” with “micro enterprise-level digital transformation keywords”. It empirically analyzes the relationship between digital transformation and innovation output in the manufacturing industry. Furthermore, it tests the related hypotheses from the perspective of internal and external transaction costs through a mechanism analysis. On this basis, the study also analyzes the nonlinear impact of digital transformation on innovation output under different levels of market competitiveness and market freedom.

The findings of this study are as follows:

(1) The development of digital transformation significantly promotes overall innovation output, as well as the quality and quantity of innovation output in the manufacturing sector.

(2) One of the mechanisms through which digital transformation affects innovation output is by reducing internal transaction costs such as management, advertising, and sales. Another mechanism is by reducing external search and innovation recombination costs in the innovation process, enhancing the efficiency of external transactions for manufacturing companies.

(3) The study also found that the intensity of the impact of digital transformation on innovation output differs under different external environmental conditions, namely, varying levels of market competitiveness and market freedom level. As market competitiveness increases, the promoting effect of digital transformation on innovation output weakens. On the other hand, as marketization level increases, the promoting effect of digital transformation on innovation output strengthens. Finally, considering endogeneity issues and conducting various robustness tests, the conclusion that digital transformation promotes innovation output remains valid.

The research findings of this study provide the following policy implications for promoting the integration of macro and micro digital transformation and supporting the innovation development of manufacturing companies:

Based on the above research results, this study offers several policy implications. Firstly, at the policy-making level, governments should strive to establish diverse digital service platforms and digital ecosystems to facilitate precise connections between digital service platforms and manufacturing companies. This will enhance the external transaction efficiency of innovative entities and enable manufacturing companies to benefit from the advantages brought by macro digital transformation in a cost-effective and convenient manner. It will also allow manufacturing companies to better leverage their role as the “engine” of economic growth in the national economy. Secondly, at the micro level of enterprises, manufacturing companies should accelerate the scope of digital technology application, and integrate their own digital technology applications into the process of urban macro digital transformation. They should utilize digital service platforms to reduce internal transaction costs, streamline management processes, improve marketing and sales strategies, and seek innovative financing support. Furthermore, manufacturing enterprises have expedited the information exchange between the demand and supply sides of the innovation market by promoting the coordinated development of their own digital technology application and macro-level digital infrastructure. This has resulted in the harmonization and integration of enterprise innovation output services or products with the innovation demands of the consumer market, thereby achieving value realization of innovative goods or services in the consumer market. Ultimately, this facilitates a positive feedback loop between the innovation supply market and the innovation demand market. Thirdly, at the external environmental level, policy-makers should create a market environment conducive to enhancing transaction efficiency, monitor and address unfair competition practices by dominant market players, especially those with significant market control power. This will ensure that reasonable market competitiveness and market freedom further strengthen the promoting effect of digital transformation on innovation output. Lastly, continuous monitoring and evaluation of the impact of digital transformation on innovation output should be conducted to ensure the effectiveness of policy interventions.
